# The contribution of environmental DNA to exploring hypogeous fungal diversity and vulnerability

**DOI:** 10.1186/s12866-026-04984-y

**Published:** 2026-03-24

**Authors:** Chang Wan Seo, Shinnam Yoo, Hannah Suh, Dohye Kim, Hyun Lee, Young Woon Lim

**Affiliations:** 1 School of Biological Sciences and Institute of Biodiversity, Seoul National University, Seoul, Republic of Korea; 2 Biodiversity Research Division, National Institute of Biological Resources, Incheon, Korea

**Keywords:** Endemism, IUCN, Red list, Sequestrate fungi, Soil organism, Truffles

## Abstract

**Background:**

Hypogeous fungi play important ecological roles and have significant economic value. However, the hypogeous habit of these organisms hinders our understanding of their diversity and vulnerability.

**Results:**

The first comprehensive assessment of the hypogeous fungal diversity in Korea was conducted using environmental DNA metabarcoding, encompassing 643 soil samples collected from 162 nationwide grids. A total of 186 phylotypes of hypogeous fungi (representing 32 genera) were identified through phylogeny-based identification using a curated ITS reference database that comprised 3,359 sequences from 693 species (83 genera). This number largely exceeds the 30 species recorded in Korea. The environmental preference analysis revealed two distinct ecological clusters of hypogeous fungal genera, respectively vulnerable to climate warming and to soil eutrophication. Based on geographic range criteria (Area of Occupancy and Extent of Occurrence), 10 of 15 phylotypes exhibited distribution patterns consistent with Endangered or Vulnerable thresholds.

**Conclusions:**

These findings reveal extensive cryptic diversity, establish baseline conservation data for hypogeous fungi, and provide a replicable methodology for global hypogeous fungal assessments in soil ecosystems.

**Supplementary Information:**

The online version contains supplementary material available at 10.1186/s12866-026-04984-y.

## Background

Hypogeous fungi produce sequestrate fruit-bodies (including ascocarps, basidiocarps, and glomerocarps) buried underground [1, 2]. While the definition of hypogeous fungi varies, following Sulzbacher et al. [3] this study adopts the broad definition that encompasses all fungi producing macroscopic underground fruit-bodies [4–6]. The hypogeous habit has convergently evolved in *Ascomycota* and *Basidiomycota* [7, 8], and is also observed in *Glomeromycota* and *Mucoromycota*, representing one of the successful adaptation strategies of fungi [6, 9]. Given their diverse origins, hypogeous fungi exhibit diverse ecological strategies, though several traits are commonly associated with this group. Many hypogeous fungi play crucial roles in the ecosystems through arbuscular mycorrhizal (AM) [2] and ectomycorrhizal (EcM) symbiosis [10–13], and saprotrophic decomposition [3]. Additionally, fruit-bodies of many hypogeous fungi serve as nutrient sources for mycophagous animals [14–17]. Their economic significance is particularly evident in *Tuber*, also known as “true truffles” [18, 19], which support a multi-million euro industry through their culinary value [20, 21].

Despite their ecological and economic importance, the diversity, vulnerability, and ecology of hypogeous fungi are poorly understood due to their hypogeous habit [13]. Many hypogeous fungi rely on mycophagous animals for spore dispersal, which restricts their dispersal range [22, 23]. This strategy often results in local endemism, thereby increasing the uncertainty of diversity and vulnerability in understudied regions [24–26]. For example, 118 species of hypogeous fungi were classified as endangered in the Czech Republic and Slovakia, Denmark, Finland, Germany, the Netherlands, Norway, Poland, and Sweden under local criteria by 2001 [27]. The number is likely an underestimation, given the presence of dark taxa, limited taxonomic scope (*Ascomycota* and *Basidiomycota*), and the limited sampling capability [13].

Specialized techniques are required to investigate hypogeous fungi, as these organisms are not visible to the naked eye. For hypogeous fungi producing distinct odors, conventional methods relied upon female pigs and trained dogs to detect specific odorous volatile organic compounds [28–31]. However, the difficulties in mobilizing animals and the presence of species that emit mild or no volatile organic compounds (i.e., *Hymenogaster muticus*) hinder the utilization of odor-based methods [32]. For the hypogeous fungi that lack distinctive odors, even trained experts must conduct surveys by manually excavating the soil in small increments, a process that is both labor-intensive and inefficient. The advent of environmental DNA (eDNA) metabarcoding has emerged as a promising solution for the research of microorganisms in underground environments [33, 34]. The high-throughput and standardized nature of the metabarcoding method facilitated understanding of comprehensive diversity and ecological features in inconspicuous environments [35–38]. Since hypogeous fungi have endemic distribution patterns and elusive symbiotic relationships, eDNA metabarcoding appears well-suited for hypogeous fungi research [5, 39, 40]. Recent studies have employed the metabarcoding method to compare with fruit-body collection, with a particular focus on hypogeous fungi [37]. However, previous hypogeous fungi studies have focused on economically important taxa [41] or have limited sampling size [42].

Currently, 30 hypogeous species have been reported in the National Species List of Korea [43], including *Diehliomyces microsporus*, *Glomus* (20 species), *Mattirolomycester fezioides*, *Melanogaster tubgeriformis*, *Redeckera* (2 spp.), *Rhizopogon roseolus*, and *Tuber* (4 spp.) species. However, many hypogeous species in Korea lack molecular evidence and current occurrence data. Therefore, the hypogeous fungal diversity of Korea remains poorly understood. This research aimed to reveal the comprehensive diversity of Korean hypogeous fungi, assess the conservation status, and establish conservation strategies. To uncover phylotype-level diversity of hypogeous fungi across Korea, a phylogeny-based identification method was applied to national-scale soil eDNA metabarcoding data. Environmental drivers of genus-level hypogeous fungal abundance were assessed through correlation analysis. To evaluate the vulnerability status of each phylotype, distribution data were utilized according to the International Union for Conservation of Nature (IUCN) criteria. These findings will provide a baseline for the conservation of hypogeous fungi and establish a methodological framework for assessing hypogeous fungal diversity worldwide.

## Methods

### Soil sampling and environmental variables

**Fig. 1:**
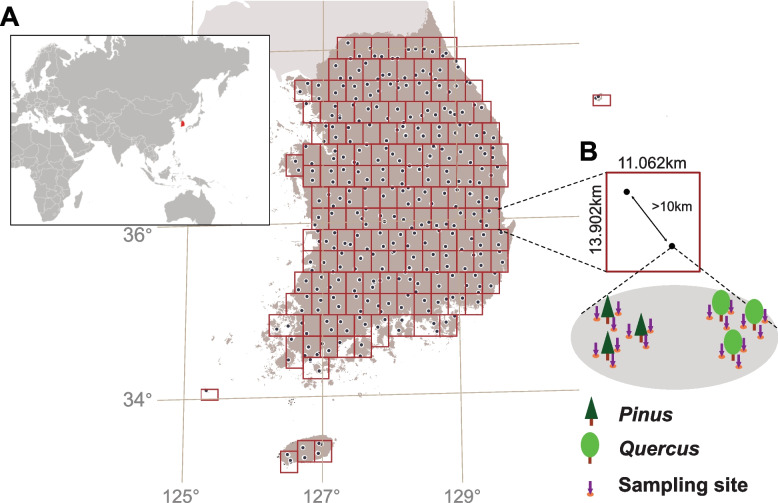
Sampling design for nationwide soil collection in South Korea. **A** Study area overview presenting the geographical location of the study region (left) and sampling grid configuration with site distribution (right). Red squares indicate sampling grids, and black dots indicate individual sampling sites. **B** Schematic diagram illustrating sampling site layout, host tree positioning, and soil replicate collection

A nationwide soil sampling was conducted across the Republic of Korea (100,210 km^2^), spanning from 33°14′25"N; 125° 6′39"E to 38°12′37"N;130°51′46"E, during 2020–2023. The research area was divided into 162 grids, each measuring approximately 11.062 km × 13.902 km, with minor adjustments made for island and coastal regions (Fig. 1; Additional file 1). Within each grid, two sites were selected at least 10 km apart, resulting in a total of 324 sampling sites. At each site, two soil samples were collected, each associated with two major EcM hosts in the Republic of Korea (*Pinus* spp. and *Quercus* spp., which account for 32% and 20%, respectively, in relative coverage) [44, 45]. The organic layer was removed, and the topsoil from 0–10 cm was collected. In total, 648 soil samples were targeted for collection across 324 sites within the 162 grids. For each host at a sampling site, soil was collected from three individual trees. From each tree, three subsamples were taken from different directions around the root zone within a 1 m radius. These nine subsamples (three trees × three directions) were then pooled into a single composite sample per host. Thus, each host species at a site contributed one pooled soil sample, which was filtered using 2 mm autoclaved sieves. Soil samples were placed on ice during transportation and stored at −80 °C in the laboratory.

The mean annual temperature (MAT) and mean annual precipitation (MAP) were obtained from the Korea Meteorological Administration using data from the nearest observatory [46]. MAT represents air temperature measured at standard height (~ 2 m aboveground); however, soil temperature at 5–15 cm depth is strongly correlated with MAT, differing by approximately −0.7 ± 2.3 °C in temperate forests [47], thus considered a proxy for underground temperature in this study. The altitude, latitude, and longitude of each site were recorded. The chemical properties of each soil sample, including pH, total organic carbon (TOC), total nitrogen (TN), ammonium ion content (NH_4_^+^), nitric acid content (NO_3_^−^), and total phosphorus (TP), were measured at the National Instrumental Center for Environmental Management (NICEM; Additional file 1).

### Environmental DNA extraction, library preparation, and sequencing

The eDNA extraction and library preparation for Illumina MiSeq sequencing were conducted by Theragen Bio (Theragen Bio Co. Ltd.). eDNA was extracted using the QIAGEN DNeasyPowerSoil® Pro Kit (Qiagen) from 0.25 g of each soil sample. The fungal internal transcribed spacer (ITS) 2 region was amplified individually from each of the 648 samples using Polymerase Chain Reaction (PCR) with the ITS3 and ITS4 primers [48], without technical replicates, under the following conditions: 95 °C/3 min, 25 cycles of 95 °C/30 s, – 55 °C/30 s, – 72 °C/30 s, and 72 °C/5 min. Each PCR product was indexed with Nextera XT Index Kit v2 (Illumina Inc., Sandiego, USA) under the same thermal conditions with 8 cycles. Library preparation was conducted using the Nextera XT DNA Library Preparation Kit v3 (Illumina Inc., Sandiego, USA), targeting 300 × 2 bp amplicon libraries. Library quality was validated using the Agilent TapeStation 4200 (Agilent Technologies, Santa Clara, CA, USA). Illumina MiSeq sequencing was carried out by the respective companies responsible for library preparation.

### Bioinformatics and hypogeous fungal identification

The raw reads from eDNA were trimmed using Cutadapt [49] with an error rate of 0.2. Amplicon sequence variants (ASVs) were denoised using DADA2 [50] in QIIME 2 [51] with parameters “–p-trunc-q 7”, “–p-max-ee-f 3”, and “–p-max-ee-r 6”. The quality filtering included the removal of chimeric sequences (“pooled” option in QIIME 2 DADA2), singleton filtering (“filter-seqs” function in QIIME 2), and ITS region validation with ITSx [52].

A three-step approach was employed to precisely identify hypogeous fungal ASVs among the total ASVs. First, a list of hypogeous genera corresponding to fungal ASV data was prepared. All genera corresponding to ASVs were extracted by querying ASVs against the NCBI Nucleotide collection database [53] using the MegaBLAST(-task megablast, -max_hsps 1, -max_target_seqs 50) [54]. The retrieved hypogenous genera and their confamilial genera were verified through literature review, excluding genera with epigeous, semi-hypogeous, and mixed habits (Additional file 2). Next, a custom ITS reference database was constructed by integrating the hypogeous sequences from published literature and the UNITE v10 dynamic database [55] for general taxonomic assignment. Database curation included nomenclature standardization using the Fungal Names Database v2024.10.22 [56] and MycoBank v2025.01.13 [57], ITS region adequacy validation with GenMine [58], removing short sequences (5.8S regions < 140 bp) with ITSx, and removing misannotated sequences with iterative FunVIP analysis (Additional file 3) [59]. Finally, phylotype-level (pt.) identification of hypogeous fungi was performed through an integrated workflow. The initial classification employed the naïve Bayes algorithm via q2-feature-classifier [60]. The ASVs from hypogeous and confamilial genera were then subjected to phylogenetic refinement using the FunVIP v0.4.0 fast mode (–search mmseqs, –outgroupoffset 1, –allow-innertrimming). Long-branch attraction artifacts were removed using TreeShrink v1.3.9 [61] or manual inspection for trees with an insufficient number of tips. The final species-level identification was confirmed using the FunVIP accurate mode (–search mmseqs, –bootstrap 100). The relative abundance of each taxon was calculated as the percentage of ASV counts relative to the total ASV counts across all samples.

### Ecological preference analysis

The relationship between hypogeous fungal genera and environmental variables was examined through statistical analysis using robust centered log-ratio (rclr) transformed genus-level ASV counts, with Wilcoxon’s rank sum test on categorical variables, and Spearman’s correlation test on numerical variables. Rclr transformation was performed on the genus-level table using the Gemelli library [62]. Host preference was evaluated with Wilcoxon’s rank sum test in the Statmodels library [63]. The correlation between each genus and numerical environmental variables (altitude, latitude, longitude, MAT, MAP, NH^4+^, NO^3−^, pH, TOC, TN, and TP) was tested with Spearman’s correlation using Statmodels library and corrected with False Discovery Rate (FDR). Hierarchical clustering of genera was performed with the linkage and dendrogram function in Scipy library (complete linkage, squared Euclidean distance) [64]. Trophic mode for each hypogeous genus was obtained from FungalTraits 1.0.0 [65].

### Vulnerability categorization

Conservation status of hypogeous fungal phylotypes was provisionally evaluated using IUCN Red List Criterion B1 (Extent of Occurrence, EOO) and B2 (Area of Occupancy, AOO) [66]. EOO was calculated using the alphashape library [67]. AOO was calculated based on 2 km × 2 km grid cells following IUCN standards, with scale correction factors applied for grid size standardization (Additional file 1) [66]. Estimated population sizes for each phylotype were estimated by adapting a metabarcoding-based approach from previous research [68] for ASV-based pooled soil samples. The formula *N =* Σ (ASVᵢ × 10 × 12/7) was applied, where ASVᵢ represents the number of ASV types per phylotype in sample i, 10 is the mature individual multiplier for fungi [68, 69], and 12/7 accounts for the average number of trees contributing to each pooled sample. Phylotypes with estimated population size exceeding 500 were assigned to vulnerability categories (Critically Endangered [CR], Endangered [EN], Vulnerable [VU], Near Threatened [NT], or Least Concern [LC]) based on spatial distribution thresholds, while those below 500 were classified as Data Deficient [DD] [70]. When EOO and AOO assessments yielded different categories, the higher threat category was assigned [66]. As complete IUCN categorization under Criterion B requires at least two of three additional subcriteria (a: severely fragmented or limited locations; b: continuing decline; c: extreme fluctuations) that cannot be assessed from single-timepoint eDNA surveys, our assessments represent potential conservation concern based on geographic range alone rather than definitive IUCN classifications. The resulting conservation assessments were compared with 33 hypogeous fungal species in the IUCN Red List [71].

## Results

### ITS database of hypogeous fungi

NCBI searches and literature verification identified 90 potential hypogeous genera across *Ascomycota*, *Basidiomycota*, *Glomeromycota*, and *Mucoromycota*. Among these, five genera (*Endogone*, *Hydnangium*, *Protubera*, *Pterosporomyces*, and *Temperantia*) lacked reliable ITS sequences, and two genera (*Horakiella* and *Sclerogone*) were illegitimate. After their exclusion, 83 genera were validated as hypogeous genera (Additional file 4). For the database construction for the validated hypogeous genera, ITS sequences were obtained from two sources. A review of existing literature (134 publications) yielded a total of 2,798 hypogeous sequences, representing 629 species. The UNITE database contributed 561 hypogeous sequences representing 416 species. The combination of these sources yielded a total of 3,359 hypogeous sequences, classified into 83 validated genera and 693 species. The final curated database also included non-hypogeous sequences to provide taxonomic context, totaling 39,935 ITS sequences (Table 1).

**Table 1:** List of hypogeous fungi found in Korean soil metabarcoding data

Phylum	Class	Order	Family	Genus	ASV	Phylotypes	Named species	Abundance (%)
*Ascomycota*	*Eurotiomycetes*	*Eurotiales*	*Elaphomycetaceae*	*Elaphomyces*	32	24	6	1.11E-01
	*Pezizomycetes*	*Pezizales*	*Discinaceae*	*Hydnotrya*	25	3	0	2.35E-02
			*Morchellaceae*	*Leucangium*	1	1	1	1.07E-04
			*Pezizaceae*	*Delastria*	33	1	0	1.23E-01
				*Hydnobolites*	11	6	1	5.14E-03
				*Luteoamylascus*	5	4	0	2.48E-03
				*Pachyphlodes*	22	8	3	8.73E-03
				*Terfezia*	8	2	0	1.31E-02
			*Pyronemataceae*	*Genea*	10	4	3	3.40E-03
				*Geopora*	1	1	1	3.91E-04
			*Tuberaceae*	*Tuber*	98	21	8	1.21E-01
		*Pezizomycetes* *incertaesedis*	*Tarzettaceae*	*Densocarpa*	1	1	0	4.67E-06
				*Paurocotylis*	2	2	0	6.48E-05
*Basidiomycota*	*Agaricomycetes*	*Agaricales*	*Hymenogastraceae*	*Hymenogaster*	57	21	8	7.46E-02
			*Stephanosporaceae*	*Stephanospora*	29	10	0	1.27E-03
		*Boletales*	*Boletaceae*	*Heliogaster*	1	1	1	2.10E-04
				*Octaviania*	2	2	1	8.51E-04
			*Coniophoraceae*	*Sedecula*	3	2	0	3.06E-04
			*Paxillaceae*	*Alpova*	21	4	1	2.18E-02
				*Melanogaster*	13	6	2	5.61E-03
				*Neoalpova*	2	2	0	3.67E-04
			*Rhizopogonaceae*	*Rhizopogon*	48	13	5	3.57E-01
			*Sclerogastraceae*	*Sclerogaster*	13	5	0	1.05E-02
		*Gomphales*	*Gomphaceae*	*Gautieria*	8	5	1	1.27E-02
		*Hysterangiales*	*Hysterangiaceae*	*Hysterangium*	1	1	0	1.05E-04
			*Trappeaceae*	*Restingomyces*	1	1	0	1.27E-04
		*Russulales*	*Albatrellaceae*	*Leucogaster*	1	1	0	5.09E-05
				*Leucophleps*	1	1	0	5.41E-04
*Glomeromycota*	*Glomeromycetes*	*Diversisporales*	*Diversisporaceae*	*Redeckera*	2	2	0	5.29E-05
		*Glomerales*	*Glomeraceae*	*Glomus*	14	11	1	6.50E-04
				*Sclerocarpum*	1	1	0	8.97E-06
*Mucoromycota*	*Endogonomycetes*	*Endogonales*	*Endogonaceae*	*Jimgerdemannia*	56	19	2	2.66E-03

### Taxonomic diversity of hypogeous fungi in Korea

**Fig. 2:**
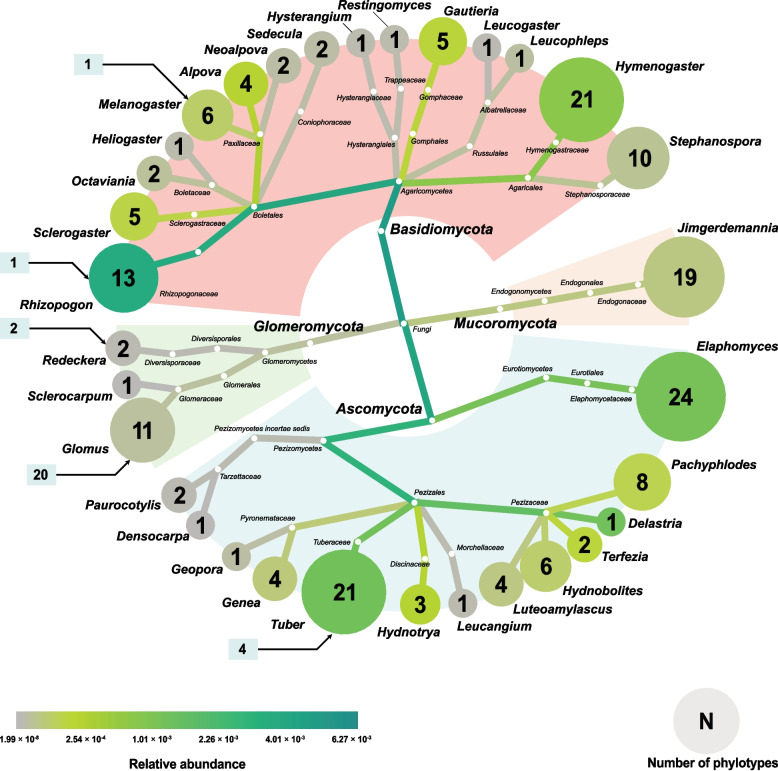
Heat tree indicating the diversity of hypogeous fungi in Korea. The color of each node indicates the relative abundance of genera within the entire sample, and the size of each tip and the numbers inside the tips indicate the number of phylotypes found within each genus. The numbers in the sky-blue boxes indicate the number of species belonging to each genus within the Korean species checklist

Of the 648 soil samples initially planned, five were excluded: two because host tree colonies were absent at the designated sites (4–41-01-S and 4–41-02-S), and three due to insufficient sequencing yields (3–01-01-H, 3–10-01-S, and 4–10-01-S). As a result, a total of 643 soil samples were included in the final analysis (Additional file 1). Sequencing of these samples generated 65,203 ASVs, of which 523 ASVs (0.80%) corresponded to 186 hypogeous phylotypes (Additional file 5). These phylotypes were distributed across 32 genera (Table 1, Fig. 2). Within this set, 45 phylotypes could be assigned to described species, while the remaining 141 were classified as dark taxa.

Most of the hypogeous fungi detected belonged to *Ascomycota* or *Basidiomycota*. *Rhizopogon* exhibited the highest relative abundance (0.36%), followed by *Delastria* (0.12%), *Tuber* (0.12%), and *Elaphomyces* (0.11%) (Table 1). The remaining hypogeous fungal genera accounted for less than 0.1% of the total abundance. *Densocarpa* (4.67 × 10^–6^%) was the rarest hypogeous fungal genus in Korea.

The presence of hypogeous genera was observed in 591 out of 643 samples (91.9%), and 161 out of 162 grids (99.4%). Gageodo Island (grid 4–41: 34°4′29"N 125°6′39"E) was the only grid without hypogeous genera. The mean abundance of hypogeous fungal genera was 0.90% (SD: 1.99%), with slightly lower abundance in *Pinus* soils (0.80%, SD: 2.08%) compared to *Quercus* soils (0.98%, SD: 1.89%). The relative abundance of hypogeous genera comprised up to 26.63% in *Pinus* soil samples and 23.90% in *Quercus* soil samples.

### Environmental preference of hypogeous fungal genera

**Fig. 3:**
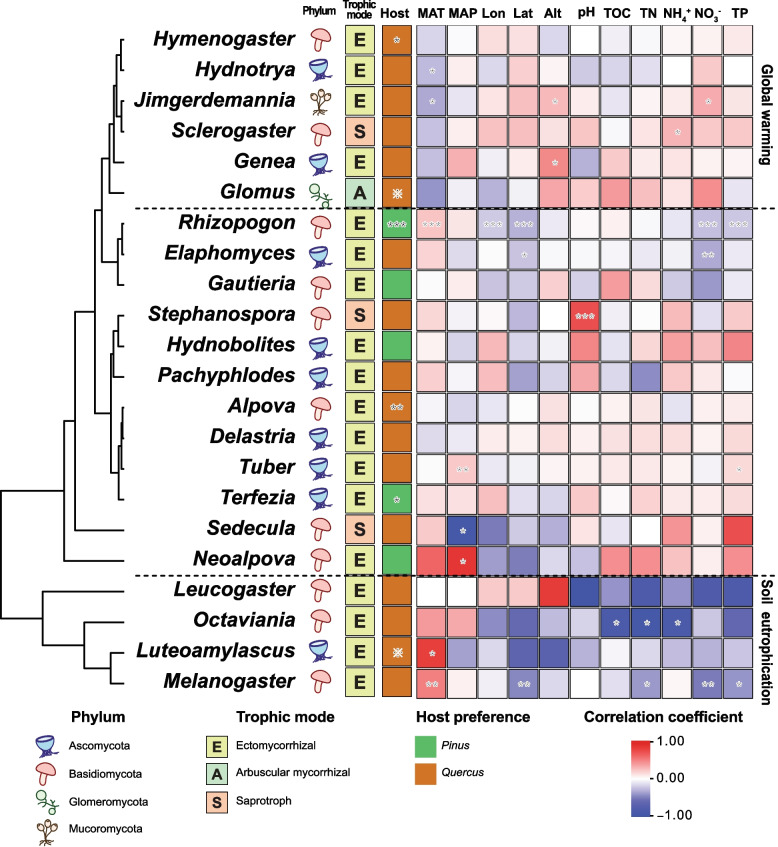
Correlation test and host preference test for hypogeous genera and environmental variables. The dendrogram on the left side of the figure represents hierarchical clustering results of environmental variable preferences. The right side of the figure represents the host preference test and correlation test with numerical variables that have undergone FDR correction. The colors used for the numerical tests indicate the correlation coefficient, while the colors for the host preference tests indicate the genera preferred by each host. Asterisks within each cell denote the statistical significance of the test (*: *p < *0.05, **: *p < *0.01, ***: *p < *0.001). Reference marks (※) denote that statistical tests cannot be performed, but ASVs only exist in the specific host. Host: Host preference, MAT: mean annual temperature, MAP: mean annual precipitation, Lon: longitude, Lat: latitude, Alt: altitude, pH: pH, TOC: total organic carbon, TN: total nitrogen, NH_4_^+^: ammonium ion content, NO_3_^−^: nitric acid content, TP: total phosphorus

*Glomus* and *Luteoamylascus* were exclusively detected in *Quercus* soil. However, statistical analysis (rclr) was not feasible, as they were identified in only one host. Host specificity and environmental correlations were analyzed for 21 of the 32 detected hypogeous genera, while the remaining 11 were excluded due to their occurrence in a single grid. Among EcM genera, *Rhizopogon* (*p =* 0.000276) and *Terfezia* (*p =* 0.0295) exhibited a significant association with *Pinus* spp., while *Alpova* (*p =* 0.00539) and *Hymenogaster* (*p =* 0.0134) exhibited a significant association with *Quercus* spp. (Fig. 3). Saprotrophic genera exhibited no significant host preferences.

Hierarchical clustering of environmental factor preferences yielded two distinct ecological clusters. The first cluster included six genera: *Genea*, *Glomus*, *Hydnotrya, Hymenogaster*, *Jimgerdemannia,* and *Sclerogaster*. These genera showed vulnerability to global warming, which was reflected in their negative correlations with mean annual temperature (MAT). Within this cluster, *Hydnotrya* (ρ = −0.265, *p =* 0.0154) and *Jimgerdemannia* (ρ = −0.326, *p =* 0.012) exhibited a significant negative correlation with MAT. Most genera within this cluster exhibited positive correlation with altitude and latitude. *Genea* (ρ = 0.450, *p =* 0.0407) and *Jimgerdemannia* (ρ = 0.266, *p =* 0.0421) exhibited a significant positive correlation with altitude.

The second cluster comprised four genera (*Leucogaster*, *Luteoamylascus, Melanogaster,* and *Octaviania*) that exhibited negative correlations with soil fertility indicators. These genera exhibited negative correlations with TOC, TN, NH_4_^+^, NO_3_^−^, and TP. *Octaviania* exhibited a significant negative correlation with TOC (ρ = −0.841, *p =* 0.0361), TN (ρ = −0.899, *p =* 0.0149), and NH_4_^+^ (ρ = −0.841, *p =* 0.0361), while *Melanogaster* exhibited a significant negative correlation with TN (ρ = −0.377, *p =* 0.0280), NO_3_^−^ (ρ = −0.499, *p =* 0.00268), and TP (ρ = −0.400, *p =* 0.0202). Notably, this cluster comprised exclusively EcM fungi and exhibited negative correlations with soil pH.

### Vulnerability of hypogeous fungi

**Fig. 4:**
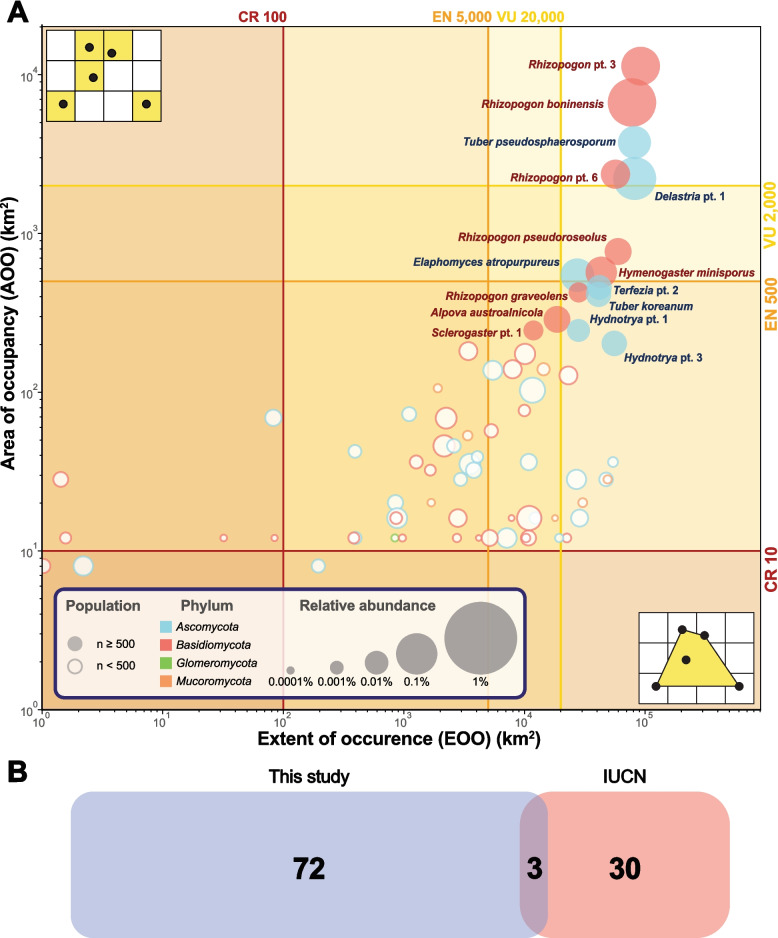
**A** Scatter plot of hypogeous phylotypes in terms of AOO and EOO values. Phylotypes with EOO greater than 0 were exclusively displayed in the dot plot. Colored lines in the plot indicate IUCN Red List Criteria thresholds (CR: Critically Endangered, EN: Endangered, VU: Vulnerable). The shape of the marker indicates whether the population size of the phylotype exceeds the IUCN Red List Criteria threshold (*n =* 500). The color of each marker and annotation label indicates the phylum of the phylotype. The size of the marker indicates the relative abundance of the phylotype in the Korean soil metabarcoding data. **B** Venn diagram showing hypogeous phylotypes between Korean soil metabarcoding data and the IUCN Red List

To assess the vulnerability of 186 phylotypes, distribution metrics (AOO and EOO) were calculated (Additional file 5). EOO values could not be determined for 111 phylotypes restricted to only one or two grids. Of the remaining 75 phylotypes, 15 had estimated population sizes exceeding 500 (Fig. 4A). Five of these (*Delastria* pt. 1, *Rh. boninensis*, *Rhizopogon* pt. 3, *Rhizopogon* pt. 6, and *T. pseudosphaerosporum*) exhibited distribution patterns consistent with LC thresholds, reflecting both widespread occurrence and high abundance. The other ten phylotypes exhibited distribution patterns consistent with threatened categories: Two (*Alpova austroalnicola* and *Sclerogaster* pt. 1) met VU thresholds, and the other eight (*Elaphomyces atropurpureus, Hydnotrya* pt. 1*, Hydnotrya* pt. 3*, Hymenogaster minisporus, Rh. graveolens, Rh. pseudoroseolus, Terfezia* pt. 2*,* and *T. koreanum*) met EN thresholds.

Comparison of our vulnerability assessment with the IUCN Red List revealed both concordance and discrepancies (Fig. 4B). Of the 33 hypogeous fungal species currently listed in the IUCN Red List, three species were detected in our mycobiome data. Two species matched exactly (*A*. *austroalnicola* and *Heliogaster columellifer*), while one (*Rh. yakushimensis*) corresponded to closely related phylotypes (*Rhizopogon* pt. 6, *Rhizopogon* pt. 7, and *Rhizopogon* pt. 8). *Alpova austroalnicola* (IUCN Red List: VU) met the EN threshold based on distribution metrics. *Heliogaster columellifer* (IUCN Red List: LC) was classified as DD due to its population size falling below the detection threshold. *Rhizopogon* pt. 6 met the LC threshold, whereas *Rhizopogon* pt. 7 and *Rhizopogon* pt. 8 could not be evaluated because of their insufficient population size. The remaining 31 species listed in the IUCN Red List were not detected in Korean soil samples.

## Discussion

### Hidden hypogeous fungal diversity of Korea extensively exceeds specimen-based records

Our eDNA metabarcoding analysis revealed a strikingly higher diversity of hypogeous fungi compared with the 30 species in seven genera documented from fruit-body derived records in Korea. Specifically, 186 phylotypes representing 32 genera were detected, of which 25 genera were EcM fungi. Among the EcM genera, *Elaphomyces*, *Hymenogaster*, *Jimgerdemannia*, *Rhizopogon*, and *Tuber* exhibited high phylotype diversity. These genera are known to form ectomycorrhizal associations with *Pinus* and *Quercus*, and their diverse representation in this study suggests they may be important components of hypogeous fungal communities in Korean forests [72–75]. In terms of relative abundance, four genera (*Delastria*, *Rhizopogon*, *Elaphomyces*, and *Tuber*) dominated the Korean soil mycobiome, each exceeding 0.1%. Notably, *Delastria*, although represented by only one phylotype, ranked second in abundance and is known as an EcM associate of *Carpinus*, *Cistus*, *Helianthemum*, *Pinus*, *Quercus*, and *Tuberaria* in Mediterranean and North American deserts [76]. Accordingly, as eDNA of these EcM hypogeous fungi was frequently detected across Korean forest soils associated with *Pinus*, *Quercus*, further investigation is warranted to understand their ecological roles in these ecosystems.

Within the *Glomeromycota*, three genera of arbuscular mycorrhizal (AM) fungi were detected, including *Glomus*, which exhibited notable diversity with 11 phylotypes. These AM fungi are globally recognized for their associations with diverse host plants [77, 78] and form dual mycorrhization patterns in EcM trees alongside EcM fungi [79, 80]. However, AM fungi research in Korea has primarily concentrated on economically important crops, with EcM trees remaining understudied [81, 82]. This limited research focus on the ecological characteristics of AM fungi may explain the high diversity observed in this study, which differs substantially from the previously reported AM fungi species in Korea. In contrast, saprotrophic fungi represented minor abundance and diversity, comprising six genera (*Densocarpa*, *Paurocotylis*, *Restingomyces*, *Sclerogaster*, *Sedecula*, and *Stephanospora*). Among these well-known decomposers in forest soils, *Sclerogaster* and *Stephanospora* exhibited notable diversity [7].

The recorded hypogeous fungi in Korea to date are restricted to the following groups: (1) taxa producing subepigeous fruit-bodies that are relatively easy to detect in forest environments, such as *Rhizopogon* [83], (2) taxa with economic value, such as *Tuber* [84, 85], *Melanogaster tuberiformis* [86], and *Mattirolomyces terfezioides* [87], and (3) taxa that show a relatively high number of records in Korea largely due to the presence of experts, such as *Glomus* and *Redeckera* [88–90]. Of the 30 recorded species, only five were confirmed by eDNA metabarcoding. The low detection rate can be attributed to the lack of comparable reference sequences for 11 species, and the reclassification of seven species into non-hypogeous genera, placing them outside the scope of this study. European species *Rhizopogon roseolus*, *Melanogaster tuberiformis*, and *Tuber uncinatum*—which were originally reported based on morphology alone [86, 91]—were not confirmed in this study. Recent studies have shown that Asian EcM fungi, although morphologically similar to European and North American counterparts, frequently represent distinct lineages [92–94]. Thus, morphological resemblance to European species likely accounts for their misidentification in Korea.

The total diversity of hypogeous fungi in Korea is likely to exceed that documented in this study, though estimates are subject to several methodological considerations. The study focused on soils surrounding the most prevalent EcM hosts in Korea, *Pinus* and *Quercus* species, to ensure uniform sampling across grids. While this approach enabled comprehensive, broad-scale coverage, the diversity of hypogeous fungi associated with alternative host species may have been underrepresented. Many hypogeous fungi may interact with plant hosts beyond *Pinus* and *Quercus*, and the present survey could not capture their diversity. For instance, *Mattirolomyces terfezioides* is known to associate with *Robinia pseudoacacia* [87]. Furthermore, selecting the appropriate genetic markers is crucial for detecting and identifying hypogeous fungi in eDNA metabarcoding. The ITS region may fail to detect or identify certain hypogeous fungi in the analysis, such as *Endogone* and *Jimgerdemannia*, due to its low specificity to universal primers [95]. The ITS region alone may provide inaccurate species delimitation, especially for unrecorded phylotypes, such as *Rhizopogon* pt. 1–8, and should therefore be interpreted cautiously. Genera exhibiting mixed habits, such as *Cortinarius* [96], were excluded to prevent dark taxa with unknown habits from being misclassified as hypogeous fungi. Conversely, our adoption of a broad definition of hypogeous fungi may yield higher diversity estimates compared with studies using narrower definitions limited to *Ascomycota* and *Basidiomycota*. Although the three genera of *Glomeromycota* (*Glomus, Redeckera, Sclerocarpum*) and one genus of *Mucoromycota (Jimgerdemannia)* included in this study were all verified through literature to produce hypogeous fruit-bodies [2, 6, 14, 97, 98], some studies do not classify these taxa as hypogeous fungi [5]. Nevertheless, the eDNA-based approach has effectively revealed the extent of previously unrecognized diversity and highlighted critical gaps in knowledge of hypogeous fungi. As most of the confirmed taxa are EcM fungi, they may represent indigenous species in East Asia.

### Environmental preferences analysis

The analysis of environmental preference revealed consistent patterns with the previously reported ecological characteristics of hypogeous fungi. These include the host specificity of *Rhizopogon* species to *Pinus* [99] and the preference of *Tuber* species for high pH soils [100]. These validations support the reliability of our ecological interpretations for other genera that have received limited academic attention. Based on environmental preference analysis and hierarchical clustering, two clusters of hypogeous genera were detected: the global warming vulnerable cluster and the soil eutrophication vulnerable cluster. Regarding global warming impacts, *Genea* and *Jimgerdemannia* exhibited significant positive correlations with altitude and appear particularly at risk because high-altitude environments restrict further upward migration under warming conditions [101, 102]. Conversely, genera exhibiting positive correlations with MAT, such as *Luteoamylascus*, *Melanogaster*, and *Rhizopogon*, may experience habitat expansion under warming scenarios, potentially facilitating the establishment of species previously restricted to tropical regions [103]. However, the limited distribution of ectomycorrhizal hosts in tropical and subtropical regions [104], combined with the extremely limited hypogeous fungal research in tropical and subtropical areas, makes it difficult to evaluate such range expansion potential [83]. This potential for climate-driven range expansion is supported by documented cases, such as the successful establishment of *T. melanosporum* in previously unsuitable northern European habitats [105].

Regarding the soil eutrophication vulnerable cluster, these genera exhibited negative correlations with TOC, TN, NH^4+^, NO^3−^, and TP, indicating vulnerability to soil eutrophication. The eutrophication of forest soil can result from atmospheric nitrogen deposition from air pollution and highland agriculture, having the capacity to modify underground EcM fungal communities [106–111]. This cluster consisted exclusively of EcM fungi, which are adapted for nutrient acquisition under oligotrophic conditions and exhibit reduced competitiveness in fertilized environments [112].

### Vulnerable hypogeous fungi

Research on hypogeous fungal diversity has advanced in scattered locations where taxonomic expertise exists, such as Australia and Europe [27, 39, 113]. For economically important taxa such as *Tuber*, commercial potential has attracted broader international research attention, including research on climate change-driven distribution shifts in the UK and Thailand [114, 115]. However, substantial regions, including Korea, lack sufficient data for conservation assessment, particularly for hypogeous fungi without recognized economic value. Despite these data limitations, the research successfully categorized the vulnerability levels of hypogeous fungi in understudied areas. The present study has indicated that the current vulnerability categorization of hypogeous fungal species in the IUCN Red List may be overestimated. For instance, *Elaphomyces atropurpureus* [74] and *Alpova austroalnicola* [116] have been reported only from Spain and South America, respectively, yet they have been discovered in Korea. This suggests that these species have broader geographic ranges. Conversely, the analysis identified eight previously unassessed phylotypes that were categorized as VU or EN, suggesting that a significant proportion of DD or unassessed species may be at risk of conservation threats.

Additionally, while potential vulnerability of hypogeous phylotypes was evaluated based on geographic range criteria (B1 and B2) using eDNA data, complete IUCN categorization requires meeting at least two of three additional subcriteria: (a) severely fragmented or existence in limited locations, (b) continuing decline, or (c) extreme fluctuation (IUCN 2024). Current methodological limitations hinder the application of these criteria through soil metabarcoding. Habitat fragmentation analysis remains problematic, as the 500 km threshold for fungi [69] conflicts with evidence showing population divergence at just 7.8 km [23]. Temporal dynamics assessment (subcriteria (b) and (c)) requires chronological sampling to detect population trends and fluctuations. Furthermore, the population size estimation approach adapted from a previous study [68], which applies the mature individual multiplier originally developed for fruit-body-based surveys to metabarcoding data, has yet to reach consensus within the mycological community. The relationship between ASV counts and actual fungal population size remains uncertain, and our threshold of 500 individuals for assessment eligibility should be interpreted with caution. Consequently, despite numerous taxa showing concerns in environmental preference analysis, most phylotypes cannot be categorized using IUCN criteria due to limited estimated population size and lack of chronological data.

The limited dispersal capacity of hypogeous fungi [23] makes them vulnerable to rapid environmental changes [117, 118]. However, the cryptic nature of hypogeous fungi poses a significant challenge in the detection of their population decline, necessitating urgent data acquisition and utilization. Therefore, continuous data for population trend and habitat fragmentation analysis should be acquired for complete categorization [66]. Yet, current sampling efforts remain insufficient. The Global Soil Mycobiome consortium data contains only 127,263 soil samples [119]—far below the 1,500,000 samples recommended for vulnerability assessment [120]. Moreover, specimen data remain severely limited, which would provide direct evidence for their existence. Therefore, the collection of fruit-bodies and detailed taxonomic studies should follow the results of eDNA-based analyses. As climate change and environmental pollution intensify, hypogeous fungal conservation requires coordinated research and policy initiatives to address critical knowledge gaps.

## Conclusions

The soil eDNA metabarcoding approach revealed extensive taxonomic diversity of hypogeous fungi previously neglected by traditional morphology-based research. As environmental pressures dramatically intensify, establishing effective conservation strategies for these fungi is urgent. In this study, two main drivers—global warming and soil eutrophication—were identified, providing a basis forgenus-specific conservation approach. Additionally, vulnerability categorization based on distribution analysis detected hypogeous fungi with high conservation priority at the phylotype level. However, because the current study was limited to Korea, a single time point, and insufficient data for categorization of the remaining hundreds of phylotypes, more data are demanded for hypogeous fungal conservation. Thus, spatiotemporal data on hypogeous fungi from both metabarcoding and fruit- body-based approaches are required. To address current data deficiencies, systematic training of taxonomists is needed, as conservation success depends on extensive field surveys, reliable species delimitation, and ecological understanding. Therefore, increased scientific investment and institutional support are essential to advance our knowledge of these cryptic yet ecologically important fungi.

## Supplementary Information

Additional file 1. Soil chemical properties, climatic variables, and geographical information for sampling sites.

Additional file 2. Hypogeous fungal genera validation results for reference database construction.

Additional file 3. Taxonomic information and reference sequence sources of hypogeous taxa.

Additional file 4. Curated ITS reference database for hypogeous fungal identification.

Additional file 5. Representative sequences, AOO, and EOO of hypogeous fungal phylotypes in Korea.

## Data Availability

Raw metabarcoding data used in this study are available at the NCBI Sequence Read Archive (https://www.ncbi.nlm.nih.gov/) under BioProject ID PRJNA1335892.

## Abbreviations

ITS: Internal Transcribed Spacer
IUCN: International Union for Conservation of Nature
EcM: Ectomycorrhizal
MAT: Mean Annual Temperature
MAP: Mean Annual Precipitation
TOC: Total Organic Carbon
TN: Total Nitrogen
NH₄⁺: Ammonium Ion Content
NO₃⁻: Nitric Acid Content
TP: Total Phosphorus
eDNA: Environmental DNA
NCBI: National Center for Biotechnology Information
PCR: Polymerase Chain Reaction
NICEM: National Instrumental Center for Environmental Management
ASV: Amplicon Sequence Variant
pt.: Phylotype
rclr: Robust Centered Log-Ratio
FDR: False Discovery Rate
EOO: Extent of Occurrence
AOO: Area of Occupancy
CR: Critically Endangered
EN: Endangered
VU: Vulnerable
NT: Near Threatened
LC: Least Concern
DD: Data Deficient
AM: Arbuscular Mycorrhizal
